# Transportation barriers, emergency department use, and mortality risk among us adults: a national health interview survey analysis

**DOI:** 10.1186/s12889-025-24670-4

**Published:** 2025-10-06

**Authors:** Fangyuan Chen, Ryan D. Nipp, Xuesong Han, Zhiyuan Zheng, K. Robin Yabroff, Tianci Wang, Changchuan Jiang

**Affiliations:** 1 School of Medicine, Tsinghua University, Beijing, China; 2 OU Health Stephenson Cancer Center, University of Oklahoma Health Sciences Center, Oklahoma City, OK USA; 3 Surveillance and Health Equity Science, American Cancer Society, Atlanta, GA USA; 4 Anne Burnett Marion School of Medicine at Texas Christian University, Fort Worth, TX USA; 5 Division of Hematology and Oncology, Department of Internal Medicine, UT Southwestern Medical Center, Dallas, TX USA

**Keywords:** Transportation barrier, Mortality risk, ED visit, Chronic health conditions

## Abstract

**Objectives:**

To estimate the prevalence of transportation barriers to care among patients with common chronic health conditions and examine associations between transportation barriers with the outcomes of emergency department use and all-cause mortality risk using a large, nationally representative cohort in the United States.

**Methods:**

We used weighted logistic and Cox regression to assess the association of transportation barriers, ED use and all-cause mortality, in 440,249 US adults aged 18–79 from the 2002–2018 National Health Interview Survey (NHIS) and linked mortality data.

**Results:**

Transportation barrier was higher in adults with chronic health conditions (history of hypertension, cardiac diseases, chronic obstructive pulmonary disease (COPD)/asthma, class III obesity, diabetes, and cancer). It was also associated with increased risk of emergency department use (age 18–64: odds ratio (OR) = 1.45, 95% confidence interval (CI):1.41–1.50; age 65+: OR = 1.34, 95%CI:1.26–1.42), and all-cause mortality (age 18–64: hazard ratio (HR) = 1.36, 95%CI:1.24–1.49; age 65+: HR = 1.30, 95%CI:1.15–1.47). These associations also hold in subgroups with all chronic health conditions, except older adults with cancer history.

**Conclusions:**

Transportation barriers disproportionally burdened adults with chronic health conditions, which warrant effective screening for and interventions against transportation barriers for individuals with chronic diseases.

**Supplementary Information:**

The online version contains supplementary material available at 10.1186/s12889-025-24670-4.

## Background

Access to healthcare, especially timely emergency medical care, is a critical determinant of health outcomes [1, 2]. The Centers for Medicare and Medicaid Services (CMS) has called for increased efforts to study and address barriers in accessing care in order to reduce health disparities and improve patient outcomes [1]. However, for millions of Americans, particularly those with chronic health conditions, lack of reliable, safe, and affordable transportation poses a significant obstacle to receiving timely and essential care [3, 4]. In a previous study, approximately 5.8 million adults in the United States reported delaying medical care within the past 12 months alone due to a lack of transportation, highlighting the importance of further research on transportation barriers to care [4]. 

Vulnerable groups, such as individuals with lower household incomes, those from racial and ethnic minority communities, with older ages, living in rural areas, and with disabilities, bear a disproportionate burden of transportation barriers to care [4–6]. These barriers exacerbate existing health inequities by limiting access to preventive care, disease management, and timely treatment, thereby leading to increased reliance on emergency department (ER) use and potentially avoidable hospitalizations [7–10]. Individuals with chronic conditions often require regular and frequent healthcare visits and ongoing disease management, and thus transportation barriers to care can be especially detrimental for these patients, including worsening health, increased risk of treatment complications, and premature mortality [8, 11, 12]. 

While previous studies have documented the prevalence and predictors of transportation barriers on healthcare utilization and outcomes in patients with certain health conditions, such as cancer or heart failure [8, 12], limited studies have comprehensively examined the association between transportation barriers and mortality risk in the general population, and among patients with any type of common chronic condition. In addition, the generalizability of findings derived from patients with cancer or heart failure to those with other chronic conditions and the general population remains unclear, as patients with cancer or heart failure need longer-term chronic disease management and/or may have higher mortality risk than the general population, respectively.

To fill this gap, in the current study, we sought to estimate the prevalence of transportation barriers to care among patients with common chronic health conditions and examine associations between transportation barriers, ED use and all-cause mortality risk using a large, nationally representative cohort. Findings from this work have the potential to inform the development of targeted interventions and policies to improve transportation access, reduce health disparities, and ultimately, improve health outcomes [1]. 

## Methods

### Study participants

Study participants were identified from the 2002–2018 National Health Interview Survey (NHIS) and NHIS Linked Mortality Files [13, 14]. The NHIS is an annual, cross-sectional, nationally representative in-person survey of the civilian, non-institutionalized population of the United States. It was conducted by trained U.S. Census Bureau field staff using a multistage probability sampling design. One adult (≥ 18 years) per family was randomly selected for the Sample Adult interview, which was conducted facetoface using computerassisted personal interviewing (CAPI), where interviewers read questions and entered responses into a laptop. The annual final response rate of NHIS was approximately 60% of the eligible adults during the study period (the number of completed Sample Adult interviews divided by the total number of eligible sample adults, then multiplying the conditional rate by the overall family response rate). We included adults who had valid vital status as of December 31, 2019. We only included adults aged 18–79 years because the NHIS does not provide single year of age for adults aged 80 years or older, but groups them in a single age category, limiting survival analysis. We excluded participants who had missing information on key conditions (*N* = 9,290), education attainment (*N* = 1,929), health insurance coverage (*N* = 1,468), functional limitations (*N* = 325), and those with missing data on whether they had transportation barriers to care (*N* = 344). (Supplemental Figure A).

In the NHIS, participants were asked at the time of the survey whether a doctor or other health professional had ever told them that they had any health condition(s), including arthritis, cancer, chronic obstructive lung disease or asthma, class III obesity (estimated by self-reported body mass index (BMI) ≥ 40 kg/m^2^ or BMI ≥ 35 kg/m^2^ with an obesity-related health condition such as any arthritis, diabetes, heart disease, and hypertension), cardiac disease, diabetes, hypertension, kidney disease, liver diseases, and stroke.

## Exposure

Consistent with previous studies [4, 5, 8], delays in care due to transportation barriers were measured by a question during the in-person NHIS interviews, “Have you delayed getting care in the past 12 months because you did not have transportation?”.

## Outcomes

We measured ED usage as patient-reported number of ED usage during the past 12 months preceding the survey, via the question, “During the past 12 months, how many times have you gone to a hospital emergency department about your own health?” Participants were classified as having “ED use” or “no ED use” based on their responses.

The NHIS Linked Mortality files were used to measure vital status for NHIS respondents through December 31, 2019, which provided 1–18 years of follow-up. Quarter and year of death was available for respondents who died during the study period, which included vital status (alive or deceased), quarter and year of death for decedents, and grouped underlying causes of death. These person-level files are linked via unique identifiers and include linkage eligibility metadata, which allowed precise calculation of time-to-event using either the interview quarter/year or the death quarter/year.

## Covariates

Covariates were chosen based on previous research examining transportation barriers [3–8, 10, 11], and existing knowledge on risk factors for poor outcomes, including age (years at survey), sex (male, female), race/ethnicity (Hispanic, Non-Hispanic White, Non-Hispanic Black, other races/ethnicities), region (Midwest, Northeast, South, West), marital status (married and non-married), education (less than high school, high school graduate, some college and more), functional limitations, family income, health insurance (Age ≤ 64: any private, public only, uninsured/missing; Age > 64: Medicare and private, Medicare Advantage/HMO, Medicare and Medicaid, Medicare only/other), and comorbid conditions (grouped as 0, 1, 2, and ≥ 3). Functional limitations were measured from a series of questions about difficulty doing specific activities due to health issues, such as shopping, walking a quarter mile, or walking up ten steps without resting. Family income was measured by ratio of family income to the poverty threshold, categorized as < 100%, 100–199%, 200–399%, and ≥ 400%. The number of comorbid conditions was based on a series of questions on self-reported health conditions as described above. This study included a long time-period, and thus we included survey era (2000–2004, 2005–2009, 2010–2014, and 2015–2018) as one of the covariates to account for economic and other secular trends.

### Statistical analysis

All analyses incorporated NHIS sampling weights and accounted for the survey’s complex, multistage sampling design using the svydesign function in the survey package in R, with parameters specified as id = ~ PSU_P (primary sampling unit), strata = ~ STRAT_P (sampling strata), and weight = ~ SA_WGT_NEW (final sampling weight for adults), and nest = TRUE to indicate that PSUs are nested within strata. Descriptive analyses were stratified by age group (18–64 and 65–79 years) to reflect differences in employment and age-eligibility for Medicare coverage. The prevalence of delays in care due to transportation barriers by age group and health condition using Chi-square tests. To further examine the interaction between age and health insurance, we presented the estimated prevalence of transportation barriers by age and health insurance among adults with each health condition.

Multivariable logistic regression was used to estimate the association between transportation barriers and ED use. After ensuring proportionality with visual inspection of log-log survival curves, we used weighted multivariable Cox proportional hazards models to examine the association of transportation barriers and all-cause mortality, adjusted for survey era, sex, race and ethnicity, region, marital status, education, functional limitations, family income, health insurance, and number of comorbid conditions. Due to the insurance difference by age group in the US, mortality risk was estimated with separate multivariable weighted Cox proportional hazards models by age group (18–64 years and 65–79 years) and health condition. Age at survey was used as the timescale in all survival analyses, which is equivalent to controlling for single year of age and recommended for analyses of household survey-mortality data linkages [8, 15]. 

As individuals with severe conditions may experience more transportation barriers to care, we performed a sensitivity analysis of excluding patients who passed away within the first two years from the initial interview to mitigate potential effects of reverse causality.

For statistical analyses, we used SAS statistical software, version 9.4 (SAS Institute Inc.) and R version 4.1.2 (‘survey’ package (v4.2-1) and ‘gtsummary’ package (v1.7.2)), adopting survey weights to account for the complex design of the NHIS linked Mortality File and survey nonresponse [13, 14]. All statistical significance testing was 2-sided at *p* < 0.05. Institutional review board approvals were exempted by University of Texas Southwestern Human Research Protection Program, as the data were deidentified and obtained from a publicly available database.

## Results

### Participant characteristics and transportation barriers to care

**Fig. 1:**
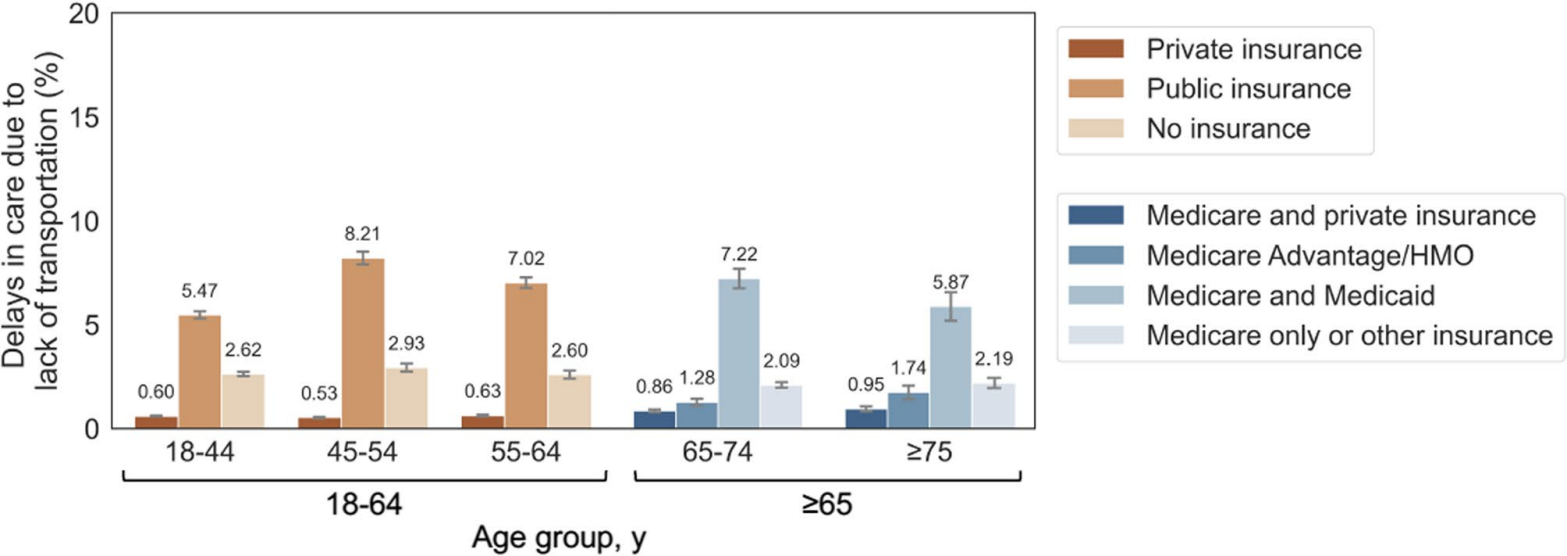
Notes: [Public insurance included Medicare, Medicaid, State Children's Health Insurance Program, and/or other public hospital/physician coverage. Age ≤64 y public insurance comprised people younger than 65 years who had one or more types of public coverage and did not have private coverage. Age ≥ 65 y Medicare and Private, comprised people age 65 and older who had Medicare and private insurance coverage, and did not have Medicare Advantage or HMO. Age ≥ 65 y Medicare Advantage/HMO, comprised people age 65 and older who had Medicare Advantage or HMO and did not have Medicaid coverage. Age ≥ 65 y Medicare and Medicaid, those 65 years or older who had both Medicare and Medicaid coverage. Age ≥ 65 y Medicare only or other, comprised people age 65 and older who had Medicare only and/or one or more of other types of public coverage except for Medicaid or no coverage. Average percentage was labeled on top of each bar. Error bars indicate 95% CIs]

We included a total of 368,330 and 71,949 adults aged 18–64 years and 65–79 years, respectively, in our cohort. Delays in care due to transportation barriers in the 12 months prior to the survey were reported by 1.8% of younger adults (*N* = 8,050) and 1.8% of older adults (*N* = 1,566), respectively. Transportation barriers to care were more common among women, non-Hispanic Black or Hispanic individuals, and individuals who were unmarried, uninsured or with public insurance, with functional limitations, lower educational attainment, and higher comorbidity burden in both age groups (Table 1; Fig. 1, Supplemental Figure B).

**Table 1:** US adults’ self-reported transportation barrier to care by age group, data from NHIS 2002–2018

	Adults of 18–64 years Delays in care due to lack of transportation *N* (%): 368,300 (86.9)			Adults of 65–79 years Delays in care due to lack of transportation *N* (%): 71,949 (13.1)		
Total (weighted %)	No Hardship *%	Hardship %	*p*-value †	No Hardship %	Hardship %	*p*-value
	No *n* = 360,250 (98.2)	Yes *n* = 8050 (1.8)		No *n* = 70,383 (98.2)	Yes *n* = 1566 (1.8)	
Age, y						
18–44	59.5	57.3	0.006	-	-	-
45–54	22.4	23.3		-	-	
55–64	18.1	19.4		-	-	
65–74	-	-	-	75.3	74.7	0.7
75–79	-	-		24.7	25.3	
Sex						
Male	49.3	37.3	< 0.001	45.1	29.4	< 0.001
Female	50.7	62.7		54.9	70.6	
Race and ethnicity						
Hispanic	15.6	19.9		8.1	15.6	
Non-Hispanic Black	12.3	24.2		9.4	20.2	
Non-Hispanic White	65.9	50.4		78.0	58.1	
Other races and ethnicities‡	6.1	5.5		4.5	6.1	
Current marital status						
Married	54.8	25.4	< 0.001	62.0	28.6	< 0.001
Not married§	45.2	74.6		38.0	71.4	
Ratio of family income to the poverty threshold						
< 100%	11.1	42.7	< 0.001	7.3	27.1	< 0.001
100–199%	14.9	25.5		17.1	32.3	
200–399%	26.1	14.5		27.8	17.8	
≥ 400%	36.8	7.9		30	7.4	
Unknown	11.2	9.4		17.9	15.5	
Health insurance coverage||						
Age ≤ 64, any private	68.9	22.8	< 0.001	-	-	-
Age ≤ 64, public only	13.2	50.0		-	-	
Age ≤ 64, uninsured or missing	17.8	27.3		-	-	
Age > 64, Medicare and private	-	-	-	50.2	24.9	< 0.001
Age > 64, Medicare Advantage/HMO	-	-		13.3	10.4	
Age > 64, Medicare and Medicaid	-	-		7.1	29.3	
Age > 64, Medicare only or other	-	-		29.3	35.4	
Any functional limitations¶						
No	73.1	33.0	< 0.001	40.7	9.0	< 0.001
Yes	26.9	67.0		59.3	91.0	
Education						
Less than high school	12.8	31.1	< 0.001	19.4	40.4	< 0.001
High school graduate	26.2	31.3		30.7	28.6	
Some college or more	61.0	37.6		49.9	31.0	
Number of comorbid conditions**						
0	60.8	35.6	< 0.001	16.8	5.6	< 0.001
1	20.9	19.8		24.6	12.4	
2	10.1	15.0		25.1	19.3	
≥ 3	8.2	29.6		33.5	62.6	
Region						
Midwest	23.5	24.2	< 0.001	22.9	22.9	0.003
Northeast	17.6	14.2		19.3	14.3	
South	36.5	39.4		37.3	41.3	
West	22.4	22.3		20.5	21.4	
Survey eras						
2002–2004	16.7	12.2	< 0.001	14.8	13.5	0.040
2005–2009	28.9	28.9		26.0	24.6	
2010–2014	30.0	32.1		30.4	28.7	
2015–2019	24.4	26.7		28.8	33.2	
Emergency department use (past 12 months)						
No	81.1	50.3	< 0.001	79.3	55.5	< 0.001
Yes	18.9	49.7		20.7	44.5	
Emergency department visit times (past 12 months)						
0	81.1	50.3	< 0.001	79.3	55.5	< 0.001
1	12.3	19.0		13.6	20.2	
2	4.8	17.1		5.4	17.2	
≥ 3	1.8	13.6		1.7	7.2	

*HMO* Health Maintenance Organization, *COPD* chronic obstructive pulmonary disease

* %

† chi-squared test with Rao & Scott’s second-order correction

‡ Other race and ethnicity includes Native American and Alaska Natives, multiple races, and unknown race and/or ethnicity

§ Not married includes widowed, divorced, separated, or never married

|| Public insurance included Medicare (federal insurance for adults ≥ 65 years and certain younger individuals with disabilities or end-stage renal disease), Medicaid (state-federal program for low-income individuals), State Children’s Health Insurance Program (SCHIP; coverage for children in families with incomes too high for Medicaid but too low for private insurance), and other public hospital/physician coverage (government-funded programs providing care through public hospitals or clinics). Age ≤ 64 y public insurance comprised people younger than 65 years who had one or more types of public coverage and did not have private coverage. Age ≥ 65 y Medicare and Private, comprised people age 65 and older who had Medicare and private insurance coverage, and did not have Medicare Advantage or HMO. Age ≥ 65 y Medicare Advantage/HMO, comprised people age 65 and older who had Medicare Advantage or HMO and did not have Medicaid coverage. Age ≥ 65 y Medicare and Medicaid, those 65 years or older who had both Medicare and Medicaid coverage. Age ≥ 65 y Medicare only or other, comprised people age 65 and older who had Medicare only and/or one or more of other types of public coverage except for Medicaid or no coverage

¶ Functional limitations included any self-reported limitation in walking a quarter of a mile, walking up 10 steps without resting, standing or sitting for 2 h, stooping, reaching up over head, carrying 10 pounds, pushing large objects such as a living room chair, shopping, or visiting friends

** Comorbid conditions included arthritis, cancer, chronic obstructive lung disease or asthma, class III obesity, cardiac disease, diabetes, hypertension, kidney disease, liver diseases, and stroke. cardiac disease included coronary artery diseases/heart failure/other general heart conditions

## Transportation barriers to care among patients across health conditions

**Fig. 2:**
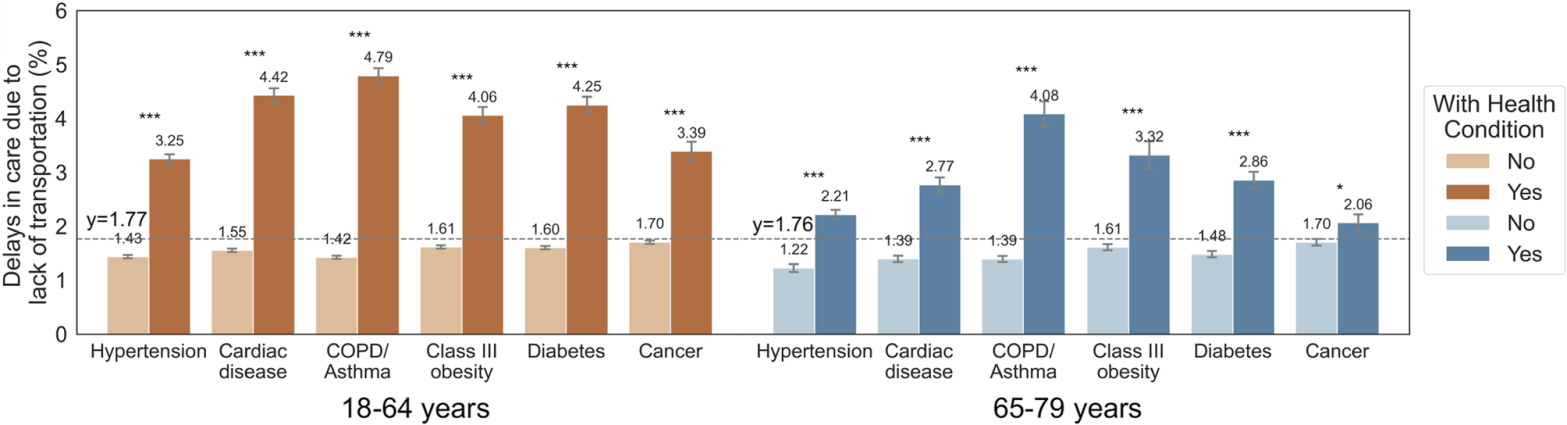
Notes: [Health conditions include hypertension, cardiac disease, COPD or asthma, class III obesity. Horizontal dashed line and y labels represent proportion of adults with patient-reported delayed care due to transportation barriers in all population of 18-64 years (1.77%) and 65-79 years (1.76%), regardless of comorbidity states. *P* value from Chi-square test with Rao & Scott’s second-order correction for complex survey samples for paired comparison *p<0.05,**p<0.01, ***p<0.001. Error bars indicate 95% CIs]

The prevalence of transportation barriers to care was higher among younger adults with hypertension (3.3% vs. 1.4%), cardiac disease (4.4% vs. 1.6%), COPD/asthma (4.8% vs. 1.4%), class III obesity (4.1% vs. 1.6%), diabetes (4.3% vs. 1.6%), and cancer history (3.4% vs. 1.7%) compared to individuals without these conditions. Similarly, older adults with hypertension (2.2% vs. 1.2%), cardiac disease (2.8% vs. 1.4%), COPD/asthma (4.1% vs. 1.4%), class III obesity (3.3% vs. 1.6%), diabetes (2.9% vs. 1.5%), and cancer history (2.1% vs. 1.7%) had higher prevalence of transportation barriers than their peers without these conditions (all *p* < 0.05; Fig. 2).

### Transportation barriers to care and emergency department use

During the study period, 16.0% of younger adults and 17.5% of older adults had at least one emergency department visit in the preceding 12 months at time of the survey, respectively. In adjusted analyses, patient-reported delayed care due to transportation barriers was associated with ED use across both age groups (18–64 years: odds ratio (OR) = 1.45, 95% Confidence Interval (CI):1.41–1.50; 65–79 years: HR = 1.34, 95%CI:1.26–1.42).

**Fig. 3:**
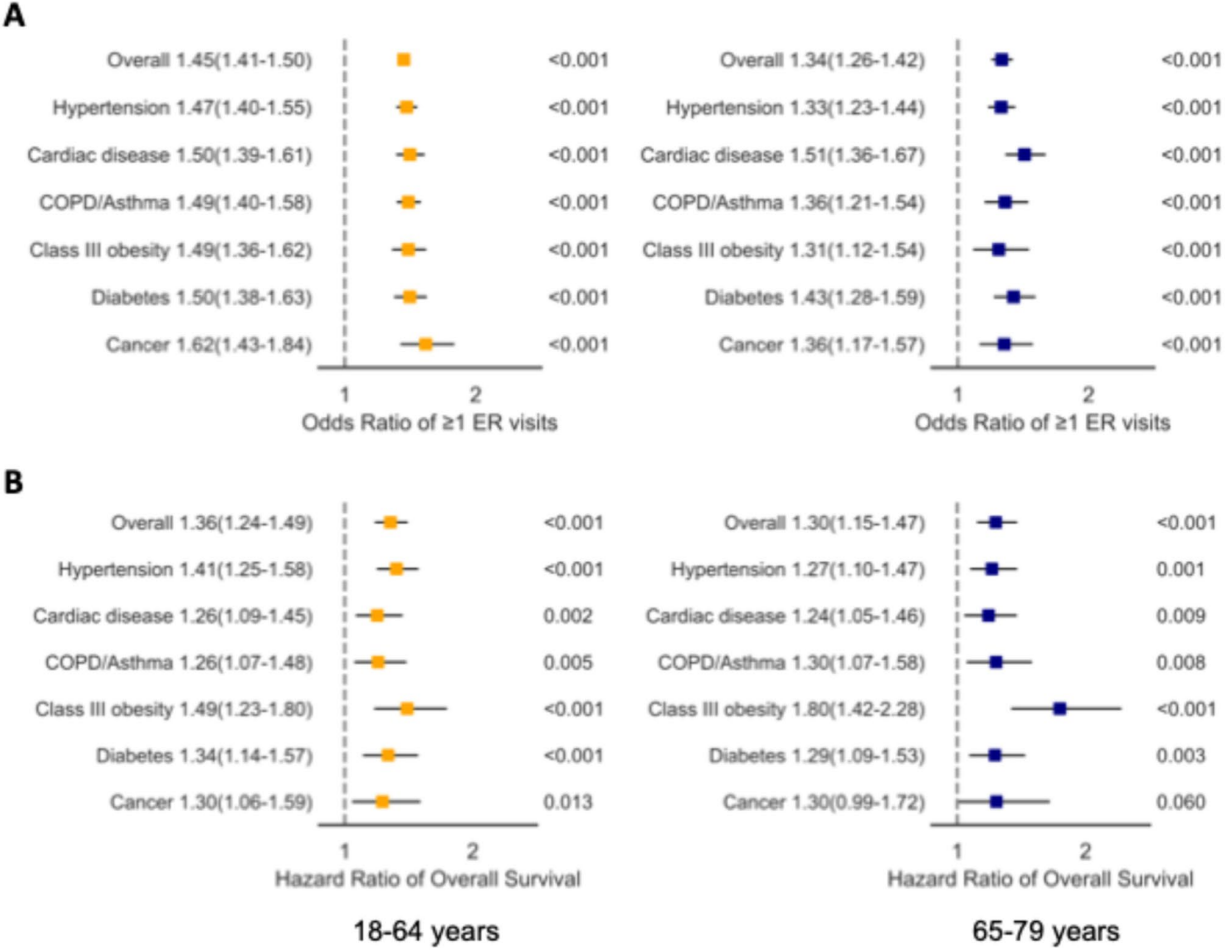
Notes: [**A**. Adjusted odds ratios were derived using separate multivariable logistic regression models of having ≥1 ED visits in adults of 18-64 years (left) and 65-79 years (right), in all population, or adults with hypertension, cardiac disease, COPD or asthma, and class III obesity. All models used age as the timescale and adjusted for survey year, age at survey, sex, race and ethnicity, region, marital status, education, functional limitations, health insurance, family income, and number of comorbid conditions. Error bars indicate 95% CIs. In multivariable analyses, statistical significance was 2-sided at *p*<0.05.**B**. Adjusted hazard ratios were derived using separate multivariable Cox regression models for adults of 18-64 years (left) and 65-79 years (right), in all population (overall), or adults with hypertension, cardiac disease, COPD or asthma, and class III obesity. All models used age as the timescale and adjusted for survey era, sex, race and ethnicity, region, marital status, education, functional limitations, family income, health insurance, and number of comorbid conditions]

In the subgroup analyses by health conditions, patient-reported transportation barriers to care were associated with increased odds of patient-reported ED visit in the preceding 12 months among individuals with all chronic conditions. In younger adults (18–64 years), these include individuals with hypertension (OR = 1.47, 95%CI:1.40–1.55), cardiac disease (OR = 1.50, 95%CI:1.39–1.61), COPD or asthma (OR = 1.49, 95%CI:1.40–1.58), class III obesity (OR = 1.49, 95%CI:1.36–1.62), diabetes (OR = 1.50, 95%CI:1.38–1.63), or history of cancer (OR = 1.62, 95%CI:1.43–1.84). For older adults, they include individuals with hypertension (OR = 1.33, 95%CI:1.23–1.44), cardiac disease (OR = 1.51, 95%CI:1.36–1.67), COPD or asthma (OR = 1.36, 95%CI:1.21–1.54), class III obesity (OR = 1.31, 95%CI:1.12–1.54), or diabetes (OR = 1.43, 95%CI:1.28–1.59), or history of cancer (OR = 1.36, 95%CI:1.17–1.57) (Fig. 3).

### Transportation barriers to care and All-Cause mortality risk

During the study period, 4.3% of younger adults and 27.0% of older adults died, respectively. In adjusted analyses, patient-reported delayed care due to transportation barriers was associated with higher mortality risk across both age groups (18–64 years: hazard ratio (HR) = 1.36, 95% Confidence Interval (CI):1.24–1.49; 65–79 years: HR = 1.30, 95%CI:1.15–1.47).

In subgroup analyses by health conditions, transportation barriers to care were associated with increased mortality risk for nearly all chronic conditions: hypertension (18–64 years: HR = 1.41, 95% CI:1.25–1.58; ≥65 years: HR = 1.27, 95% CI:1.10–1.47), cardiac disease (18–64 years: HR = 1.26, 95% CI:1.09–1.45; ≥65 years: HR = 1.24, 95% CI:1.05–1.46), COPD or asthma (18–64 years: HR = 1.26, 95% CI:1.07–1.48; ≥65 years: HR = 1.30, 95% CI:1.07–1.58), class III obesity (18–64 years: HR = 1.49, 95% CI:1.23–1.80; ≥65 years: HR = 1.80, 95% CI:1.42–2.28), diabetes (18–64 years: HR = 1.34, 95% CI:1.14–1.57; ≥65 years: HR = 1.29, 95% CI:1.09–1.53), and history of cancer (18–64 years: HR = 1.30, 95% CI:1.06–1.59; ≥65 years: HR = 1.30, 95% CI:0.99–1.72, not statistically significant) (Fig. 3).

### Sensitivity analyses

Sensitivity analysis demonstrated a largely similar prevalence of delays in care due to transportation barriers across all health conditions subgroups in both younger and older adults. Similar associations between transportation barriers and mortality were largely consistent in sensitivity analyses when excluding patients who died within the first two years after the survey (Supplemental Figure C).

## Discussion

In this nationally representative cohort study in the United States, adults with health conditions experienced more delays in care due to transportation barriers than their peers without similar conditions, with higher prevalence in socioeconomically and medically disadvantaged adults. Notably, these transportation barriers were associated with increased emergency department use and mortality risk among both younger and older adults across nearly all chronic health conditions (except for cancer in older patients). Collectively, our findings emphasize the need for targeted interventions to address transportation barriers to care, particularly among patients with chronic conditions and multimorbidity.

Multiple factors contributed to transportation barriers to healthcare. For example, lack of vehicle access, long distances or rural location, high costs, and inconvenient public transit schedules. Transportation barriers could decrease regular office-based visits and increase emergency department use. Further, they may result in delayed diagnosis, treatment, follow-up care, and ultimately, poor disease management and increased mortality risk [6–8]. Our study findings align with previous literature that has documented the substantial burden of transportation barriers on healthcare access and utilization, particularly among vulnerable communities [3, 5, 6, 10]. Our study extends this knowledge by demonstrating the consistent association between transportation barriers and all-cause mortality risk across nearly all chronic conditions and age groups, including hypertension, cardiac disease, COPD/asthma, diabetes, and class III obesity, underscoring the pervasive impact of transportation barriers on health outcomes. It is particularly worth noting that patients with chronic medical conditions are at higher risk of experiencing medical financial hardship than the general population, making it difficult for them to afford transportation to care [16]. Therefore, transportation barriers could plausibly exacerbate disparities in health outcomes for underserved patients, as they tend to be under-insured, have more chronic conditions, and encounter additional difficulties with access to care [4]. 

Solutions to help overcome transportation barriers exist, but they face significant challenges in implementation. For example, Non-Emergency Medical Transportation (NEMT) is a health insurance benefit that can help individuals overcome transportation barriers to care [17]. However, despite the mandate requiring all state Medicaid programs to provide NEMT coverage [18], Medicaid beneficiaries still face a disproportionately high burden of transportation barriers, with less than 5% of adult Medicaid beneficiaries using NEMT benefits in 2018 [19]. NEMT program users also reported concerns about reliability, as well as complex and long wait times for scheduling and authorization processes, stigma, and inadequate supply or inflexibility of NEMT providers in certain areas [19]. Healthcare professionals and researchers should identify and address these barriers by screening patients with relevant SDOH questions, increasing NEMT utilization, and potentially improving the cost-effectiveness and accessibility of these types of services, such as using modern technology-based ride sharing services [17]. 

Notably, value-based insurance design (VBID) programs offer a promising approach to address transportation barriers and other health-related social needs (HRSNs) through supplemental benefits [20, 21]. CMS has mandated that Medicare Advantage VBID plans provide at least two supplemental benefits to address beneficiaries’ HRSNs, including transportation assistance [20]. As these VBID programs gain popularity, healthcare professionals, researchers, and policymakers should evaluate the implementation and effectiveness of supplemental benefits, identify best practices, and create a conducive environment for VBID program success. This can be achieved by providing greater flexibility for plans to tailor benefits to specific needs and allocating resources for rigorous evaluation and scaling of effective interventions, ultimately improving health equity and population health outcomes [22]. 

Patient navigation programs could also play a crucial role in addressing transportation barriers and other social needs through health-related social needs screening and connecting patients in need to appropriate resources including their insurance benefits [23–25]. Patient navigators and community health workers can identify individuals with transportation barriers, conduct social needs assessments, provide referrals to community transportation services, and assist with care coordination [25]. Previous studies have shown the effectiveness of navigation-based interventions in improving healthcare access and outcomes for patients facing transportation and other health-related social needs, whereas intervention only focusing on transportation barriers often failed to deliver improvement in healthcare utilization [26–29]. For example, Navigation for Disparities and Untimely Radiation thErapy, a navigation-based multilevel intervention that was designed to include education, travel support, care discussion and follow-up has demonstrated the potential to decrease treatment delays in patients with head and neck squamous cell carcinoma [30, 31]. These findings highlight the critical need for comprehensive, navigation-based strategies addressing multiple health-related social needs, as they significantly enhance healthcare utilization and outcomes for patients with transportation barriers.

CMS has taken steps to encourage partnerships between health systems and community health workers by making health-related social needs screening and patient navigation services billable under Medicare. However, the adequacy and sustainability of these incentives for widespread adoption and maintenance of such programs remain uncertain. Importantly, the effectiveness of health-related social needs screening efforts may also depend on the extent to which private insurers follow Medicare’s lead.

### Limitations

Our study had several limitations, and the results likely underestimated the prevalence of transportation barriers. First, the NHIS did not interview Native American individuals living on tribal reservations, nor those in long-term care institutions with chronic illnesses. Second, the NHIS asked only one question about *delays in* care due to transportation barriers in the past 12 months, and thus we could not assess the prevalence of patients forgoing care altogether because of transportation barriers, and these patients may be more likely to experience adverse health outcomes due to suboptimal chronic disease management. Third, we could not evaluate associations between geographic location (e.g., urban/rural areas, distance to care facilities) and transportation barriers to care due to the lack of information in the publicly available NHIS data. Fourth, we could not conduct detailed analyses of health condition severity due to the self-reported nature of health conditions. We also could not capture the detailed treatment data for each health condition, which may confound the association between transportation barriers to care and mortality risk. Lastly, our study is subject to survival bias, as our sample included a selected group of patients by virtue of having health conditions and surviving long enough to be sampled by the NHIS and completing the survey. For instance, patients with poor-prognosis cancers, end-stage kidney disease, or advanced stage heart failure are less likely to be included in household surveys, despite more complex medical needs. Other limitations include the use of data prior to 2018, which may not reflect the current healthcare landscape; and potential discrepancies between actual and patient-reported emergency department use and transportation barriers.

## Conclusions

In conclusion, we found a high prevalence of transportation barriers to care across multiple chronic health conditions and observed novel associations of delays in care due to lack of transportation with and higher ED use and higher mortality risks in US adults, particularly among adults with chronic health conditions. These results emphasize the urgent need for screening and testing of viable interventions to address transportation barriers to care, particularly among patients with greater medical needs.

## Supplementary Information

Supplementary material 1.

## Data Availability

The datasets used and/or analysed during the current study are available from the corresponding author on reasonable request.

## Abbreviations

BMI: Body mass index
CI: Confidence Interval
CMS: Centers for Medicare and Medicaid Services
COPD: Chronic obstructive pulmonary disease
ED: Emergency department
HR: Hazard ratio
NEMT: Non-Emergency Medical Transportation
NHIS: National Health Interview Survey
OR: Odds ratio
VBID: Value-based insurance design
