# Supplementary material for: Transportation barriers, emergency department use, and mortality risk among us adults: a national health interview survey analysis
Source: BMC Public Health. 2025 Oct 6;25:3348. doi: 10.1186/s12889-025-24670-4 (PMC12502226; doi:10.1186/s12889-025-24670-4)
Supplement: Supplementary file 1 — Supplementary material 1. [file 12889_2025_24670_MOESM1_ESM.docx]

**Table of Contents**

**Supplemental** [**figure A. CONSORT diagram of participant selection from the National Health Interview Survey 2002-2018 2**](#_heading=h.gjdgxs)

**Supplemental** [**figure B. Proportion of Adults with Delayed Care due to Transportation Barriers, Grouped by Age, Health Insurance, and Health Conditions 3**](#_heading=h.30j0zll)

**Supplemental** [**figure C. Sensitivity analysis excluding individuals who died within two years from interview 4**](#_heading=h.1fob9te)

# Supplemental Figure A. CONSORT diagram of participant selection from the National Health Interview Survey 2002-2018


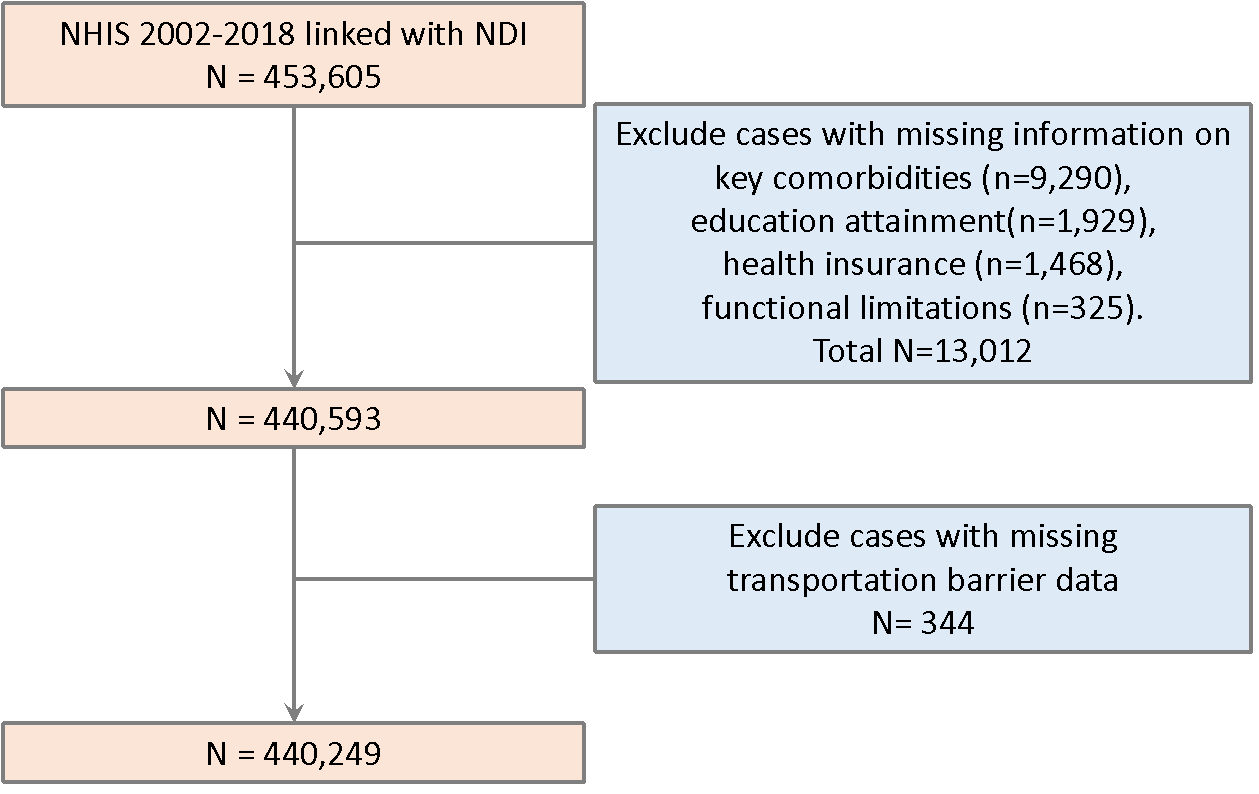


Key health conditions were based on a series of questions on self-reported health conditions, including arthritis, cancer, chronic obstructive lung disease or asthma, class III obesity, coronary heart disease, diabetes, hypertension, kidney disease, liver diseases, and stroke. Health insurance: Public insurance included Medicare, Medicaid, State Children's Health Insurance Program, and/or other public hospital/physician coverage. Age ≤64 y public insurance comprised people younger than 65 years who had one or more types of public coverage and did not have private coverage. Age ≥ 65 y Medicare and Private, comprised people age 65 and older who had Medicare and private insurance coverage, and did not have Medicare Advantage or Health Maintenance Organization (HMO). Age ≥ 65 y Medicare Advantage/HMO, comprised people age 65 and older who had Medicare Advantage or HMO and did not have Medicaid coverage. Age ≥ 65 y Medicare and Medicaid, those 65 years or older who had both Medicare and Medicaid coverage. Age ≥ 65 y Medicare only or other, comprised people age 65 and older who had Medicare only and/or one or more of other types of public coverage except for Medicaid or no coverage. Functional limitations included any self-reported limitation in walking a quarter of a mile, walking up 10 steps without resting, standing or sitting for 2 hours, stooping, reaching up over head, carrying 10 pounds, pushing large objects such as a living room chair, shopping, or visiting friends. Transportation barriers were measured by a question during the in-person interviews, “Have you delayed getting care in the past 12 months because you did not have transportation?”.

# Supplemental Figure B. Proportion of Adults with Delayed Care due to Transportation Barriers, Grouped by Age, Health Insurance, and Health Conditions

**
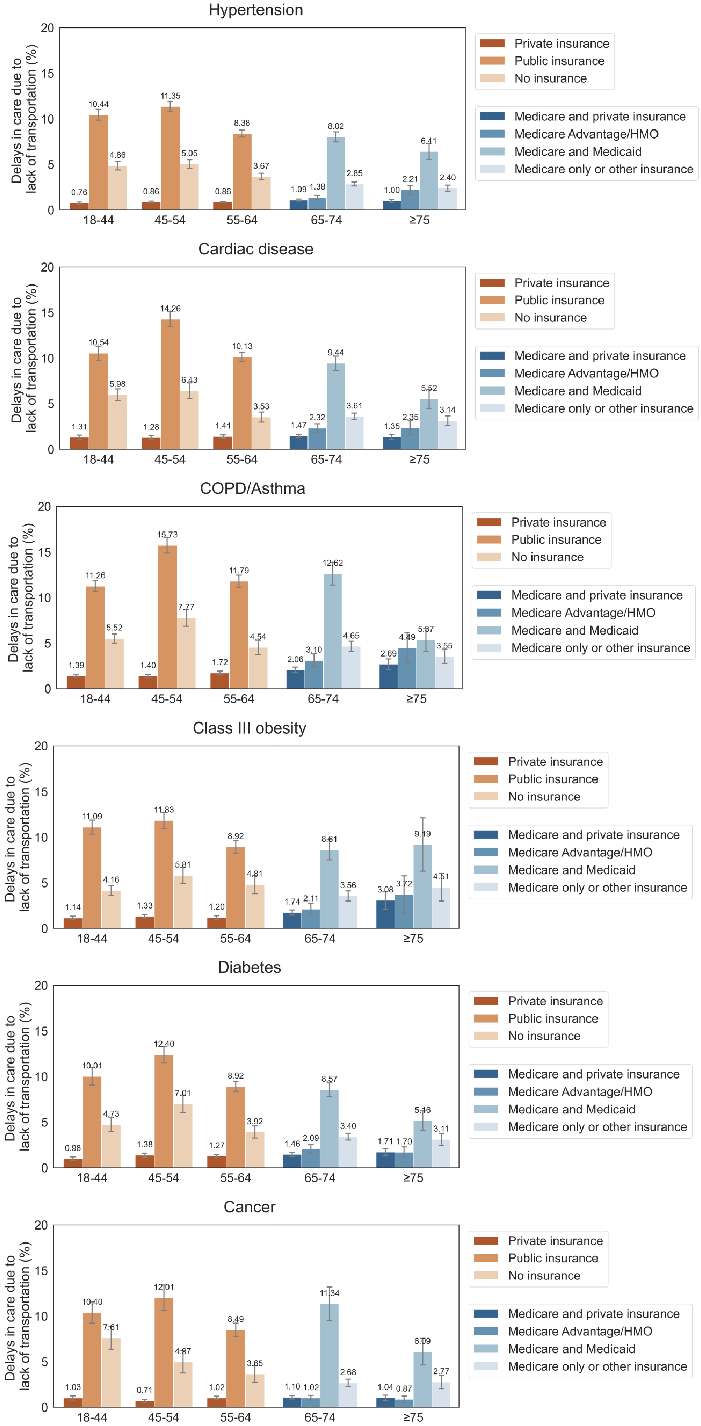
**

Health insurance: Public insurance included Medicare, Medicaid, State Children's Health Insurance Program, and/or other public hospital/physician coverage. Age ≤64 y public insurance comprised people younger than 65 years who had one or more types of public coverage and did not have private coverage. Age ≥ 65 y Medicare and Private, comprised people age 65 and older who had Medicare and private insurance coverage, and did not have Medicare Advantage or HMO. Age ≥ 65 y Medicare Advantage/HMO, comprised people age 65 and older who had Medicare Advantage or HMO and did not have Medicaid coverage. Age ≥ 65 y Medicare and Medicaid, those 65 years or older who had both Medicare and Medicaid coverage. Age ≥ 65 y Medicare only or other, comprised people age 65 and older who had Medicare only and/or one or more of other types of public coverage except for Medicaid or no coverage.

# Supplemental Figure C. Sensitivity analysis excluding individuals who died within two years from interview

**
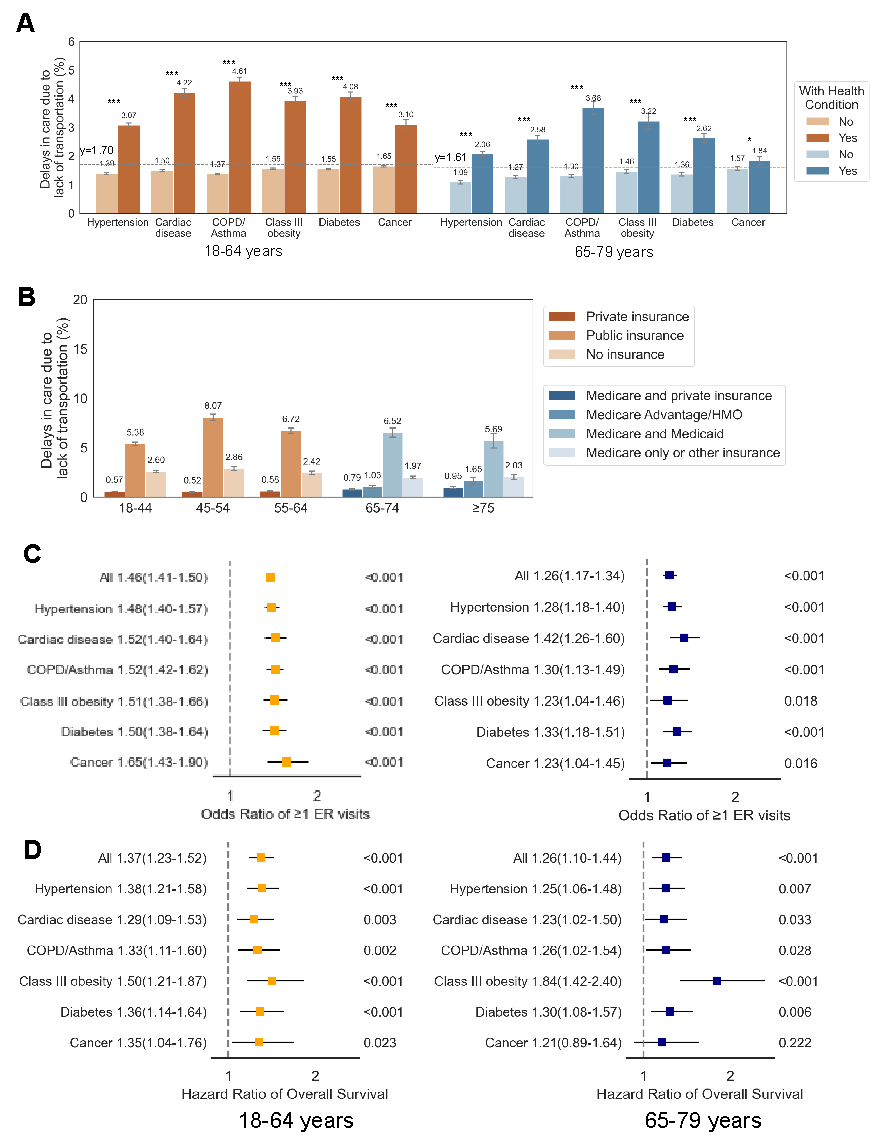
**

A. Proportion of Adults with Delayed Care due to Transportation Barriers, Grouped by Age and Insurance Status. Average percentage was labeled on top of each bar. Error bars indicate 95% CIs.

B. Proportion of Adults with Delayed Care due to Transportation Barriers, Grouped by Age and Health Conditions. Horizontal dashed line and y labels represent proportion of adults with delayed care due to transportation barriers in all population of 18-64 years (1.70%) and 65-79 years (1.61%). P value from Chi-square test with Rao & Scott’s second-order correction for complex survey samples for paired comparison *p<0.05, **p<0.01, ***p<0.001. Error bars indicate 95% CIs.

C. Adjusted odds ratios were derived using separate multivariable logistic regression models of having ≥1 ER visits in adults of 18-64 years (left) and 65-79 years (right), in all population, or adults with hypertension, cardiac disease, COPD or asthma, and class III obesity. All models used age as the timescale and adjusted for survey year, age at survey, sex, race and ethnicity, region, marital status, education, functional limitations, health insurance, family income, and number of comorbid illnesses. Error bars indicate 95% CIs. In multivariable analyses, statistical significance was 2-sided at p<0.05.

D. Overall Survival Risk of Delayed Care due to Transportation Barriers in Adults, Grouped by Age and Health Conditions. Adjusted hazard ratios were derived using separate multivariable cox regression models for adults of 18-64 years (left) and 65-79 years (right), in all population, or adults with hypertension, cardiac disease, COPD or asthma, and class III obesity. All models used age as the timescale and adjusted for survey era, sex, race and ethnicity, region, marital status, education, functional limitations, family income, health insurance, and number of comorbid illnesses.
